# Reduced hospital stay, morphine consumption, and pain intensity with local infiltration analgesia after unicompartmental knee arthroplasty: A randomized double–blind study of 40 patients

**DOI:** 10.3109/17453670902930008

**Published:** 2009-04-01

**Authors:** Per Essving, Kjell Axelsson, Jill Kjellberg, Örjan Wallgren, Anil Gupta, Anders Lundin

**Affiliations:** ^1^Department of Orthopedic SurgerySweden; ^2^Department of Anesthesiology and Intensive Care, Department of Clinical Medicine, University HospitalÖrebroSweden; ^3^University of LinkÖpingSweden

## Abstract

**Background and purpose** The degree of postoperative pain is usually moderate to severe following knee arthroplasty. We investigated the efficacy of local administration of analgesics into the operating area, both intraoperatively and postoperatively.

**Methods** 40 patients undergoing unicompartmental knee arthroplasty (UKA) were randomized into 2 groups in a double–blind study (ClinicalTrials.gov identifier: NCT00653926). In group A (active), 200 mg ropivacaine, 30 mg ketorolac, and 0.5 mg epinephrine (total volume 106 mL) were infiltrated intraoperatively into the soft tissue, while in group P (placebo), no injections were given. 21 hours postoperatively, 150 mg ropivacain, 30 mg ketorolac, and 0.1 mg epinephrine were injected intraarticularly via a catheter in group A, whereas patients in group P were injected with the same volume of saline (22 mL).

**Results** Median hospital stay was shorter in group A than in group P: 1 (1–6) days as opposed to 3 (1–6) days (p < 0.001). Postoperative pain in group A was statistically significantly lower at rest after 6 h and 27 h and on movement after 6, 12, 22, and 27 h. Morphine consumption was statistically significantly lower in group A for the first 48 h, resulting in a lower frequency of nausea, pruritus, and sedation. Postoperatively, there were improved functional scores (Oxford knee score and EQ–5D) in both groups relative to the corresponding preoperative values.

**Interpretation** Local injection of analgesics periarticularly at the end of the operation and intraarticularly at 21 h postoperatively provided excellent pain relief and earlier home discharge following UKA. There was a high degree of patient satisfaction in both groups after 6 months (Clinical Trials.gov: NCT 00653926).

Postoperative pain is often severe after knee arthroplasty (Wang et al. 2002). Traditionally, this has been managed with epidural analgesia, continuous peripheral nerve blocks, or parental opioid drugs. Although epidural analgesia is efficacious (Axelsson et al. 2005), side effects and some rare but major complications such as spinal hemorrhage and spinal infection have recently led to questioning of its routine use, specifically in older women (Moen et al. 2004). Peripheral nerve blocks provide good analgesia, but in order to control pain effectively, it may be necessary to block the sciatic, femoral, and obturator nerves (McNamee et al. 2002), which can be technically demanding. Parenteral opioids are associated with side effects. This has led to a search for new strategies in order to avoid the problems of regional analgesia or opioids, and to minimize the complications.

Recently, a local infiltration analgesia (LIA) technique was developed by Kerr and Kohan in Sydney, Australia (Röstlund and Kehlet 2007, Kerr and Kohan 2008). With this LIA technique, a long–acting local anesthetic (ropivacaine), a non–steroidal anti–inflammatory drug (ketorolac), and epinephrine are infiltrated periarticularly intraoperatively and via an intraarticular catheter postoperatively. Effective pain relief with early mobilization and reduced hospital stay was reported.

Minimally invasive techniques for arthroplasty have become increasingly popular, especially for unicompartmental knee arthroplasty (UKA) in medial non–inflammatory arthritis. Together with an intraoperative multimodal analgesia program and rapid mobilization, the minimally invasive technique is believed to reduce the hospital stay further (Beard et al. 2002, Reilly et al. 2005, Carlsson et al. 2006, Berend and Lombardi 2007). However, the specific role of the LIA technique has not been fully investigated in double–blind studies.

The main aim of this double–blind study was to evaluate whether intra- and postoperative administration of ropivacaine, ketorolac, and epinephrine into the operating field would affect the length of hospital stay. Secondary endpoints were morphine consumption, pain intensity, and side effects. In an attempt to assess the safety of the technique, knee function and patient satisfaction scores were also determined for up to 6 months after surgery.

## Patients and methods

The study protocol was approved by the regional ethics committee (May 4, 2005, EudraCt no. 2005–000685–39) and the Swedish Medical Products Agency, and conducted in accordance with the Declaration of Helsinki (ClinicalTrials.gov identifier: NCT00653926).

57 consecutive patients scheduled for unicompartmental knee arthroplasty (UKA) because of osteoarthritis were screened for eligibility. The inclusion criteria were: age 20–80 years, ASA physical status I–III, and mobility indicating normal preoperative mobilization. Exclusion criteria included known allergy or intolerance to one of the study drugs, serious liver, heart or renal disease, chronic pain, or bleeding disorder.

### Randomization and blinding

**Figure 1.**
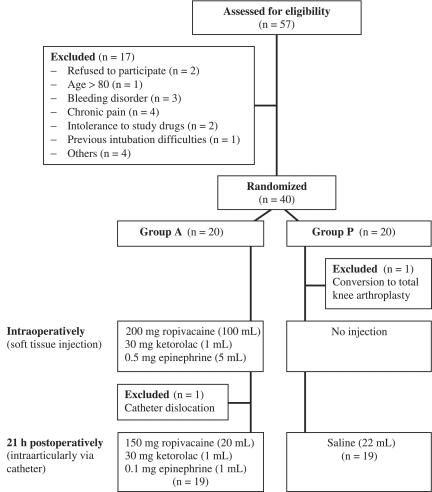
Flow chart for the study.

57 patients were assessed for eligibility and 17 were excluded prior to randomization; see the flow chart for details (Figure 1). Thus, 40 patients were enrolled in this randomized double–blind study. Written informed consent was obtained from each patient before the start of the study. Surgery was performed at the Department of Orthopedics, Örebro University Hospital from September 2005 through March 2007 and patients were followed for 6 months after surgery.

The hospital pharmacy randomized the patients into 2 groups with 20 patients in each, using computer–generated randomized numbers. Group A (active) received a multimodal injection intraoperatively and postoperatively while group P (placebo) received no injection intraoperatively and a saline injection postoperatively as detailed below. On the day before surgery or on the morning of surgery, the surgeon called the hospital pharmacy to receive the group randomization. The patients, the 2 study investigators, the study physiotherapist, and all the staff concerned with the postoperative care of the patients were blinded. Since the operating surgeons were not blinded, they did not take any part in patient care after completion of the operation.

### Anesthesia

All patients received diazepam (10 mg) orally 1 h before planned surgery and all operations were performed under general anesthesia. When designing this study, we decided not to use spinal or epidural anesthesia in order to be able to assess differences in early postoperative pain and mobilization more accurately, and to ensure blinding of the patients as to treatment. Cloxacillin (1 g) was given intravenously before surgery and at 8, 16, and 24 h postoperatively. For thrombo–prophylaxis, Dalteparin (5,000 IU) was administered subcutaneously once each evening for 10 days, starting on the evening before surgery.

### Surgery

All operations were performed using a minimally invasive technique. The same technique was used in both groups, and all patients received the Link Endo–Model Sled Prosthesis (Link Sweden AB, Akersberga, Sweden). A tourniquet was used in all patients.

### Pain management

In group A, 200 mg ropivicaine, 30 mg ketorolac, and 0.5 mg epinephrine (total volume 106 mL) were infiltrated by the surgeon into the soft tissues periarticularly during the operation in the following way. Before inserting the components, 20–30 mL was injected into the posterior capsule and before closure of the wound, the rest was injected into the capsule incision, the synovium, the ligament, and the subcutaneous tissue. In group P, no injections were given. All patients had a tunnelled intraarticular multihole 20–G catheter placed at the end of the operation by the surgeon. No drain was placed in the wound. A compression bandage and ice packs were applied during the first 6 h. A patient–controlled analgesia (PCA) pump with morphine (1–mg bolus with 6–min lockout time) was connected intravenously, which was used as rescue medication by all patients. All patients received 1 g paracetamol orally 4 times a day, starting on the preoperative morning. After 21 h, 150 mg ropivicaine, 30 mg ketorolac, and 0.1 mg epinephrine, total volume 22 mL, were injected intraarticularly via the catheter in group A and a similar volume of saline was injected in group P. Pain assessments were made using a 100–mm visual analog scale (VAS) and, at 24 h, if pain at rest was < 40 on the VAS during a 2–h period, the PCA pump was discontinued and paracetamol (1 g) and tramadol (50 mg) orally were administered up to 4 times daily as required. The intraarticular catheter was removed after 24 h and the tip of the catheter was sent for culture.

### Mobilization and home discharge

The first attempt at mobilization was made 6 h postoperatively, when the patient was encouraged to stand up and to walk 2–3 steps. If unsuccessful, mobilization was attempted again on the following day. Patients were discharged when they fulfilled the discharge criteria (see below). After discharge, the patients were asked to complete a questionnaire regarding postoperative pain on days 1, 3, and 14.

### Outcome measures

*Hospital stay.* The time to discharge (where day 0 was the day of the operation) was assessed by a physician and the study physiotherapist (who were unaware of the group randomization) according to the following criteria: mild pain (VAS <30) sufficiently controlled by oral analgesics, ability to walk with elbow crutches, ability to climb 8 stairs, ability to eat and drink, and no evidence of any surgical complication.

*Pain.* Assessment of pain (VAS) was done at 3 h, 6 h, 12 h, 21 h, 22 h (i.e. 1 h after injection of the test drug in the knee catheter), 27 h, on days 3 and 14, and at 3 and 6 months postoperatively. Pain was assessed both at rest and with motion (60 degrees of knee flexion).

*Analgesic consumption.* Morphine consumption was recorded during 0–6 h, 6–24 h, and 24–48 h postoperatively. Oral analgesic consumption was recorded during 0–24 h, 24–48 h, 48–96 h, and 96–168 h.

*Surgical outcomes.* The physiotherapist recorded the ability to walk with a walking frame 6 h postoperatively. Knee extension and flexion were assessed preoperatively, at 27 h, at discharge, on day 3, and at 3 and 6 months postoperatively. Patient satisfaction during the first 24 postoperative hours and after 7 days was rated. Oxford knee score was determined preoperatively, at 2 weeks, and 3 and 6 months postoperatively. Oxford knee score is a validated 12–item knee questionnaire that scores patients from 12 points (the best possible) to 60 points (the worst possible) (Jahromi et al. 2004). The EuroCol (EQ–5D) questionnaire was filled in preoperatively and at 6 months postoperatively. EuroCol (EQ–5D) is a standardized instrument for use as a measure of health outcome (Fransen and Edmonds 1999). It provides a single index value from 0 to 1 where 0 represents poor health and 1 represents perfect health.

All complications and adverse events were registered intraoperatively and postoperatively, as well as after discharge. Any hospital re–admissions during the 6–month follow–up period were also recorded.

### Statistics

A power analysis was done prior to the start of the study using length of hospital stay (LOS) as the primary endpoint. In a pilot study on 8 patients, the LOS for the intervention group was found to be 3.1 (SD 1.3). In a similar group of patients, operated earlier without the LIA technique, the mean LOS was 5.5 (SD 1.5) days. With an α of 0.05 and a β of 0.2, an expected reduction of 1.5 days in the treatment group, and a standard deviation of 1.5, 17 patients would be required in each group. Assuming that there would be a somewhat greater standard deviation in the control group, 20 patients were included in each group of the study.

The Mann–Whitney–U test was used for analysis of the primary endpoint (LOS) since the data were not found to be normally distributed. Results are presented as median and 95% CI. The VAS was assessed as a supportive parameter and analyzed as if it was a primary endpoint. Mann–Whitney–U test was used to assess median pain scores and the p–values were corrected using the Bonferroni–Holm method. The other secondary endpoints (morphine consumption, knee function scores, and patient satisfaction scores) were also analyzed using the Mann–Whitney–U test. Dichotomous data were analyzed using the chi–square test or Fisher’s exact test, as appropriate. A p–value of < 0.05 was considered to be statistically significant.

## Results

### Patients

Of the 40 patients enrolled in the study, 2 were excluded: 1 in group A due to catheter disconnection and 1 in group P due to intraoperative conversion to total knee arthroplasty. Thus, 38 patients completed the study. Patient characteristics were similar in both groups (Table 1).

**Table 1.** Demographic data and duration of surgery, mean (SD)

	Group A	Group P
No. of females/males	9/10	10/9
Age, years	66 (5)	64 (6)
Weight, kg	87 (11)	85 (11)
Height, cm	171 (11)	170 (12)
BMI	30 (3)	30 (1)
ASA physical status I/II	6/13	9/10
Operation time, min	92 (13)	86 (11)

Group A (active): intraoperative, periarticular infiltration;

Group P (placebo): no intraoperative infiltration.

ASA physical status I: normal health; and II: mild systemic disease.

### Primary endpoint

**Figure 2.**
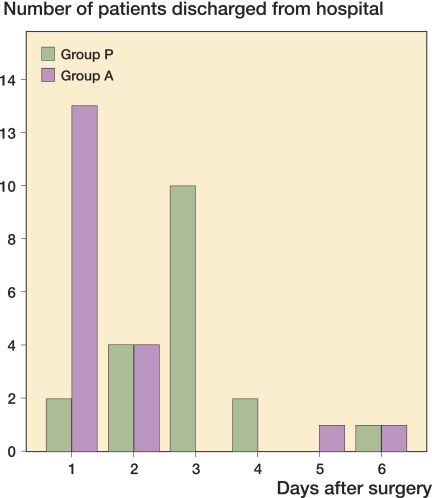
Day 1 represents the first postoperative day. Group A (active)= intraoperative, periarticular infiltration: 200 mg ropivacaine, 30mg ketorolac and 0.5 mg epinephrine; postoperative, intraarticular injection: 150 mg ropivacaine, 30 mg ketorolac and 0.1 mg epinephrine. Group P (placebo)= no intraoperative infiltration and postoperative, intraarticular injection: 22 mL saline. The median discharge times are shown: Group A = day 1 and in Group P = day 3 (p<0.001); Number of patients discharged after 2 days: 17/19 in Group A vs. 6/19 in Group P (p<001).

*Hospital stay.* Analysis of the results when including all 40 patients (intention–to–treat principle) or the 38 patients (per–protocol principle) did not reveal any statistically significant differences. Median postoperative hospital stay was less in group A (n = 19) than in group P (n = 19): 1 (1–6) days vs. 3 (1–6) days (p < 0.001), i.e. there was a median difference of 2 (CI 95% 1–2) days (Figure 2). In group A, 13/19 patients were discharged during the first postoperative day as compared to 2/19 in group P (p < 0.001). During the first 2 postoperative days, 17/19 patients were discharged in group A and only 6/19 were discharged in group P (p < 0.001). 5 patients (2 patients in group A and 3 patients in group P) had a prolonged hospital stay due to pain, and were discharged after the third postoperative day.

### Secondary endpoints

**Figure 3.**
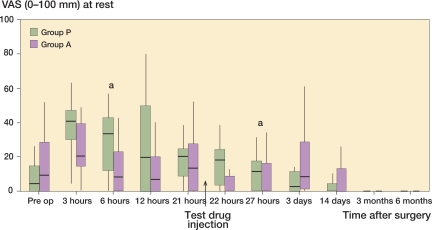
Postoperative pain at rest. VAS scores are presented as median and interquartile range (IQR). * p = 0.003 (6 h); p = 0.004 (27 h).

*Pain relief.* At rest, VAS pain score was lower in group A than in group P at 6 h (p = 0.003) and 27 h (p = 0.004) (Figure 3). At 6 months, 3 patients had registered pain in group A (5, 6, and 21) whereas in group P all patients had a VAS score of 0.

**Figure 4.**
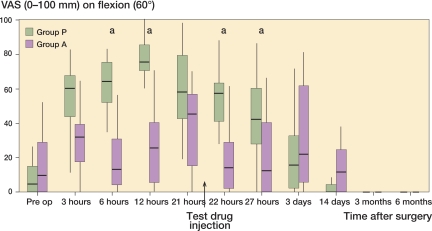
Postoperative pain on movement. VAS scores are presented as median and interquartile range (IQR). * p < 0.001 (6 h); p < 0.001 (12 h); p < 0.001 (22 h); p = 0.001 (27 h).

With movement (Figure 4), VAS pain scores were lower in group A than in group P at 6, 12, and 22 h (all with p < 0.001) and at 27 h (p = 0.001). At 6 months, all patients in both groups had a VAS score of 0.

### Consumption of analgesics

Median morphine consumption was lower in group A than in group P: 21 (0–68) mg as opposed to 67 (17–126) mg during the first 48 h postoperatively (p < 0.001). From 24 h to 48 h postoperatively, there were only 2 patients in group A that required morphine, as compared to12 patients in group P (p<0.001). There was no statistically significant difference in total tramadol consumption between the groups during the first 7 postoperative days.

### Surgical outcomes

16 of the 19 patients in group A and 9 of the 19 in group P could walk with a frame 6 h after surgery.

There was a statistically significant difference between groups A and P in knee extension and knee flexion 27 h postoperatively: 5(0–20) degrees vs. 10 (0–20) degrees (p = 0.002) for extension and 90 (75–110) degrees vs. 80 (45–100) degrees (p = 0.001) for flexion (Table 2). No statistically significant differences were found between the groups at 3 days, 3 months, and 6 months postoperatively.

**Table 2.** Mobilization and patient satisfaction

Outcome	Group A		Group P		p–value
	Median (range)	n	Median (range)	n	
Knee extension (degrees)					
preop.	5 (0–20)	19	10 (0–15)	19	0.09
27 h postop.	5 (0–20)	18	10 (0–25)	19	0.002
discharge	10 (0–20)	17	10 (0–25)	19	0.07
3 days postop.	10 (0–25)	19	10 (0–25)	19	1
3 months postop.	5 (0–15)	18	5 (0–15)	18	0.8
6 months postop.	0 (0–10)	18	0 (0–19)	18	0.7
Knee flexion (degrees)					
preop.	120 (95–135)	19	125 (90–135)	19	0.5
27 h postop.	90 (75–110)	18	80 (45–100)	19	0.001
discharge	90 (70–110)	17	90 (50–95)	19	0.5
3 days postop.	80 (60–115)	19	90 (60–95)	19	0.2
3 months postop.	120 (100–130)	18	120 (105–130)	18	0.6
6 months postop.	125 (95–135)	18	125 (105–130)	18	0.9
Patient satisfaction					
1 day postop.	4 (2–4)	19	3 (1–4)	19	0.2
7 days postop.	3 (2–4)	19	3 (1–4)	19	0.4
Oxford knee score					
preop.	40 (27–52)	19	42 (33–50)	19	0.4
14 days postop.	31 (14–44)	19	32 (14–36)	19	0.2
3 months postop.	20.5 (13–42)	19	14.5 (12–30)	18	0.01
6 months postop.	17.5 (12–35)	18	13 (12–21)	18	0.08
EQ-5D					
preop.	0.66 (0.09–0.80)	19	0.20 (–0.08–0.73)	19	0.08
6 months postop.	1 (0.66–1)	18	1 (0.8–1)	18	0.06

n: number of patients who participated varied depending on patients’ ability to cooperate. Knee extension and knee flexion: significant difference between the groups at 27 h.

Oxford knee score: 12 (the best possible) to 60 (the worst possible).

Preop. score vs. 14 days postop. scores: p = 0.05 (group A); p < 0.001 (group P).

Preop. score vs. 3-month and 6-month postop. scores: p = 0.001 (group A); p < 0.001 (groupP).

EQ-5D health outcome: 1 = perfect health; 0 = poor health; and negative values = very poor health outcome.

Preop. values vs. 6-month values: p = 0.001 (group A); p < 0.001 (group P).

The median values of patient satisfaction scores varied between 3 and 4 and there were no significant differences between the groups at 24 h and at 7 days. There was a statistically significant difference in Oxford knee score between the groups at 3 months, with higher scores in group A than in group P. No statistically significant difference in EQ–5D value was found at 6 months postoperatively.

### Adverse effects

There were no major surgical complications in any of the patients. There were higher incidences of nausea (10 vs. 4), pruritus (4 vs. 0), and sedation (4 vs. 0) in group P than in group A (p < 0.05).

There were 3 positive cultures from the catheter tips, all with coagulase–negative *Staphylococcus.* The patient in group A with positive culture had increased C–reactive protein (CRP) of short duration without any increase in leukocytes. No antibiotic therapy was given and no clinical signs of infection were found during the follow–up period. The other 2 patients in group P had no increase in CRP or leukocytes. No patients were re–admitted for any complications during the 6 months of follow–up.

## Discussion

In one recent study on total knee arthroplasty, continuous femoral nerve block was compared with the LIA technique (Toftdahl et al. 2007). The authors found reduced opioid consumption and improved pain scores in the LIA group. When correctly performed by experienced anesthesiologists, nerve blocks may be a good alternative for prolongation of postoperative pain relief. However, the simplicity of LIA and its high success rate could have an advantage over peripheral nerve blocks in this setting.

Unicompartmental arthroplasty with minimally invasive technique results in less operative trauma than total knee arthroplasty. However, moderate–to–severe pain remains a common problem. In an open pilot study (Beard et al. 2002) and a single–blind study (Reilly et al. 2005), a modified LIA technique with only infiltration of ropivacaine, ketorolac, and adrenaline intraoperatively was tested during unicompartmental arthroplasty and gave promising results. The infiltration technique in combination with oral NSAIDs and opioids resulted in a high degree of patient satisfaction, good pain relief, and early discharge with no major complications. In both studies, Redivac drain was used during the first 14–18 hours. In contrast to the above studies, we have tested the LIA technique in a double–blind study with a combination of ropivacine, ketorolac, and adrenaline administered both intraoperatively and on day 1 postoperatively without postoperative Redivac drain. Both the intraoperative infiltration and the postoperative intraarticular injection of this combination of analgesics via the catheter resulted in an acceptable degree of pain relief during the initial postoperative period; in addition, an increased range of knee movement resulted in early discharge. In addition to improved pain relief, we also found a lower consumption of rescue analgesics, which in turn resulted in a lower incidence of opioid–related side effects (including sedation, pruritus, and nausea) when LIA was used rather than placebo.

One consequence of improved pain relief is a natural improvement in mobilization. This can, however, have detrimental effects sometimes, as was seen in a study by Lombardi (Lombardi et al. 2004) on total knee arthroplasty. These authors found a “rebound effect” on postoperative pain during the first 2 postoperative days in the intervention group. A possible explanation for this finding may be higher activity levels achieved from better pain control and less sedation, which could in turn result in increased pain subsequently. Alternatively, patients may experience a delay in the onset of pain postoperatively, which can be interpreted as greater pain intensity. In our study, we could not find any evidence of such a rebound effect.

Although good analgesia can be achieved using different techniques, it is important that the time in hospital is not prolonged. Thus, our aim was to achieve good pain relief and early mobilization without prolonging recovery or hospital discharge. Since hospital discharge can be affected by several non–medical factors, we used an objective method to assesss whether the patient was ready to be sent home. These criteria have been used by other authors as a way of objectively assessing recovery and discharge (Gupta et al. 1999). Our findings show that there was a substantial reduction in hospital stay without having a negative effect on patient satisfaction. Other authors have reported similar results (Reilly et al. 2005, Berend and Lombardi 2007).

Although pain relief was better in the LIA group, this did not translate into improved knee function except at 27 h postoperatively. This can be explained in several ways. The scales used for assessment of knee function may not be sensitive enough to detect differences after knee surgery. Discharge time, used in an appropriate way, appeared to be a valuable tool. Functional assessment by Oxford knee score should have been done earlier postoperatively instead of after 2 weeks, when pain intensity was low, even in the placebo group—which may explain the absence of any differences in Oxford knee score between the groups.

We found no differences between the groups in the EuroQol preoperatively and after 6 months. Thus, data from both instruments measuring the functional assessment support the findings of others that early discharge is safe and unicompartmental techniques (Beard et al. 2002, Reilly et al. 2005, Berend and Lombardi 2007).

We used a combination of ropivacain, ketorolac, and epinephrine for LIA. It is possible that a combination of ropivacaine and ketorolac alone would have been adequate to provide a similar degree of pain relief. However, we were concerned about LA toxicity when injecting 200 mg ropivacaine locally due to its rapid absorption from the tissues and therefore added arenaline to the solution. In addition, adrenaline has been shown to have an alpha–2 agonistic effect similar to clonidine (Niemi and Breivik 2002) and we thought that we might be able to reduce pain further by using this combination.

Although the use of oral non–steroidal anti–inflammatory drugs (NSAIDs) in a multimodal analgesic regime may have reduced pain intensity further and rescue analgesic consumption, we decided not to use these for several reasons. We used ketorolac as part of the multimodal analgesic treatment with LIA, which has been found previously to be efficacious (Beard et al. 2002, Reilly et al. 2005, Toftdahl et al. 2007). However, combination of ketorolac with another oral NSAID may result in systemic toxicity. In addition, regional NSAID injections have been shown to provide better analgesia than when they are administered systemically (Reuben and Connelly 1995).

We conclude that the LIA technique can be strongly recommended for postoperative analgesia after unicompartmental knee arthroplasty. Further studies would be certainly valuable to determine the best components in the mixture of drugs to be used in the LIA technique.
